# Gingival Cyst of Newborn

**DOI:** 10.5005/jp-journals-10005-1087

**Published:** 2011-04-15

**Authors:** Aman Moda

**Affiliations:** Reader, Department of Pedodontics and Preventive Dentistry, Guru Gobind Singh College of Dental Sciences and Research Center Burhanpur, Madhya Pradesh, India

**Keywords:** Gingival cyst, Newborn.

## Abstract

Gingival cyst of newborn is an oral mucosal lesion of transient nature. Although it is very common lesion within 3 to 6 weeks of birth, it is very rare to visualize the lesion thereafter. Presented here is a case report of gingival cyst, which was visible just after 15 days of birth. Clinical diagnoses of these conditions are important in order to avoid unnecessary therapeutic procedure and provide suitable information to parents about the nature of the lesion.

## INTRODUCTION

Odontogenic cysts originate in the epithelial components of the odontogenic apparatus or from its remnants, which are entrapped within the bone or the peripheral gingival tissues. With respect to their pathogenesis, some of them are considered as “developmental” and others as “inflammatory”. Many features of the infant mouth are unique and peculiar to the birth period of development, and some benign oral mucosal conditions are frequently found in newborns, which are transient in nature. The frequency of inclusion cysts is high in newborns but they are rarely seen after 3 months of age.^1^ These cysts, also known as gingival cyst of the newborn or Bohn’s nodules, can manifest as few or many, white to yellowish, round to oval, nodes in the maxillary and/or mandibular gingiva and alveolar ridge of newborns. These nodes generally measure 2 to 3 millimeters in their largest dimension.

Fromm^2^ classified conditions affecting newborns as Epstein’s pearls, Bohn’s nodules and dental lamina cysts. Based on histological origin and location in the oral cavity, these can be classified as Epstein’s pearls, Bohn’s nodules, and gingival cysts. Gingival cysts of newborns generally occur in multiples but occasionally occur as solitary nodule also. They are located on the alveolar ridges of newborns or young infants. It is believed that fragments of dental lamina that remains within the alveolar ridge mucosa after tooth formation proliferate to form these small keratinized cysts. They are generally asymptomatic and do not produce any discomfort for the infant.^3^

Based on the location, these cysts may be divided into palatal and alveolar cysts. Those located at the mid-palatine raphe are referred as palatine cysts while those present on the buccal, lingual or crest of alveolar ridge as alveolar (or gingival) cysts.^4^ The reported prevalence of alveolar cysts in newborn ranges from 25 to 53%,^5,6^ while for palatal cysts is about 65%.^7^ Individually, the prevalence of gingival cyst of infants is 13.8%, Epstein’s pearl is 35.2% and Bohn’s nodules is 47.4% ^8^ with no sexual predilection. Although prevalence is high, these cysts are rarely seen by the dentist or pediatrician because of transient nature of these cysts, which disappears within 2 weeks to 5 months of postnatal life.

The cyst is lined by odontogenic epithelium which is covered by a thick layer of keratin, which gives the cyst its yellow color. The majority of these cysts break by themselves, a few days after birth, exuding the keratin. In some cases however, they may remain for a period of several months and in such cases surgical opening is indicated.

## CASE REPORT

**Fig. 1:**
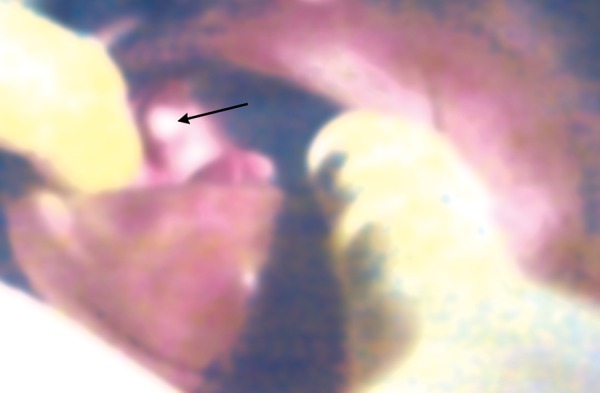
Gingival cyst of newborn

A 15-day-old child came with his mother to the department of pedodontics with a complaint of a yellowish-white swelling of gums in upper back region of the mouth. The child was full-term born and his mother gave history of presence of the lesion since birth with no contributory findings in the medical history. Intraoral examination of the child revealed a small yellowish-white nodular papule over right side alveolar ridge of posterior maxilla (Fig. 1). The size of the papule varied from 4 to 6 mm. On soft tissue examination, no other abnormality or relevant findings were found on either mucosa, tongue, palate or the floor of mouth.

On the basis of clinical examination and characteristic appearance of the lesions, a diagnosis of gingival cysts of newborn was made. Since lesions are self-limiting, the child was kept under observation and oral hygiene instructions were explained to the patient. The patient was kept on periodic recall, and the lesion had disappeared by the time patient came for his next visit after 3 months.

## DISCUSSION

Gingival cyst of newborn, also known as dental lamina cyst, is a true cyst. As it is lined by thin epithelium and shows a lumen usually filled with desquamated keratin, occasionally containing inflammatory cells.^3^ These structures originate from remnants of the dental lamina and are located in the corium below the surface epithelium. The nodes are result of cystic degeneration of epithelial rests of the dental lamina (rests of Serres). After the dental lamina invaginates to form the dental organ, the epithelial pedicle that connects the dental organ to the surface epithelium is broken down giving rise to the rests of Serres. Occasionally, they may become large enough to be clinically noticeable as discrete white swellings on the ridges. Majority of these cysts degenerate and involutes or rupture into the oral cavity within 2 weeks to 5 months of postnatal life.^9,10^ The diagnosis of gingival cyst must be differentiated from three other conditions, which are typically seen during the same time and possibly also have a location similarity. These include Natal teeth, Epstein’s pearls and Bohn’s nodules. Palatally located cysts in newborns were first described by Alois Epstein and are often referred to as Epstein’s pearls. These are also keratinized cysts which occur along the median palatal raphae and arise from the epithelium entrapped along the line of fusion. Bohn’s nodules, so called after his description of the same in 1866, are scattered over the junction of the hard and soft palate and are derived from minor salivary glands. Bohn also classified cysts in the alveolar ridges as mucous gland cysts. However, later studies by Moreillon and Schroeder^11^ have shown that these cysts are microkeratocysts.

The mechanisms behind the disappearance of the cysts in postnatal life have been attributed to a discharge of cystic keratin at the time of fusion of the cyst walls with the oral epithelium. However, it has been suggested that part of the cystic epithelium may remain inactive even in the adult gingiva. So, it becomes important that professionals involved in natal and neonatal care are able to promptly identify these cysts in order to avoid unnecessary therapeutic procedures and provide suitable information to the infant’s parents about the nature of these lesions.
